# Draft Genome Sequence of *Pseudomonas putida* Strain S610, a Seed-Borne Bacterium of Wheat

**DOI:** 10.1128/genomeA.01048-13

**Published:** 2013-12-26

**Authors:** Dongping Wang, Cliff S. Han, Armand E. K. Dichosa, Cheryl D. Gleasner, Shannon L. Johnson, Hajnalka E. Daligault, Karen W. Davenport, Po-E Li, Elizabeth A. Pierson, Leland S. Pierson

**Affiliations:** Bioscience Division, Los Alamos National Laboratory, Los Alamos, New Mexico, USAa; Departments of Plant Pathology and Microbiology, Texas A&M University, College Station, Texas, USAb; Department of Horticultural Sciences, Texas A&M University, College Station, Texas, USAc

## Abstract

We report the genome sequence of a seed-borne bacterium, *Pseudomonas putida* strain S610. The size of the draft genome sequence is approximately 4.6 Mb, which is the smallest among all *P. putida* strains sequenced to date.

## GENOME ANNOUNCEMENT

Seed-associated bacteria impact plant health and physiology in a variety of ways, affecting processes such as hormone production, nutrient acquisition, and pathogen inhibition (1, 2). These associations may occur at different developmental stages, such as during dormancy and germination in soil. *Pseudomonas putida* is a metabolically versatile and environmentally ubiquitous bacterial species (3, 4). To date, none of the seed-borne *P. putida* genomes has been sequenced.

We announce the draft genome sequence of *P. putida* strain S610, isolated from surface-sterilized wheat seeds at the Texas A&M AgriLife Research and Extension Center, College Station, TX. The isolated strain is an aggressive colonizer of both the spermosphere and the rhizosphere. Since symbiotic/commensal interactions between *P. putida* and the host plant are not well understood at a molecular level (5), an analysis of this genome will provide more insight into the specific properties related to host adaptation.

Genome sequencing was performed using a MiSeq sequencer with 250-bp read chemistry (Illumina). A modified TruSeq DNA sample prep kit version 2 and a MiSeq sequencing protocol were used during the sequencing process. Briefly, genomic DNA from strain S610 was isolated from an overnight culture using the DNeasy miniprep kit (Qiagen, Hilden, Germany). The genomic library was constructed using 1 µg of genomic DNA fragmented by the Covaris E210 instrument. The samples were then run on a gel and size selected for a range of 400 to 500 bp to accommodate the longer read length required for the instrument. The size-selected library underwent 10 cycles of PCR. The libraries were quantified using the Qubit double-stranded DNA (dsDNA) high-sensitivity (HS) assay, the Bioanalyzer high-sensitivity chip, and quantitative PCR (qPCR). A sequencing run was set up to generate 2 × 250 base-paired reads.

The filtered sequences were assembled *de novo* using SPAdes (6), wherein 69 contigs with an average of 66-fold (53- to 350-fold) genome coverage were obtained. The contigs ranged from 527 to 654,627 bp in size. The assembled data were subjected to annotation using an Ergatis workflow manager (7), and the genome size was found to be 4,596,354 bp, comprising protein-encoding genes and RNA-encoding genes. The genome is remarkably smaller than other reported *P. putida* genomes, which range in size from 5.8 to 6.9 Mb (8). The strain S610 genome harbors the genes encoding calcium-binding proteins, hemolysin, peptide transporters, multidrug efflux pumps (5), iron receptors (8), and sigma factors (9) that are potentially related to seed surface attachment and biofilm formation.

### Nucleotide sequence accession number.

This whole-genome shotgun project for *P. putida* S610 has been deposited at DDBJ/EMBL/GenBank under the accession no. AYJQ00000000.
